# Meep, a Novel Regulator of Insulin Signaling, Supports Development and Insulin Sensitivity via Maintenance of Protein Homeostasis in *Drosophila melanogaster*

**DOI:** 10.1534/g3.120.401688

**Published:** 2020-09-30

**Authors:** Matthew T. Pereira, Katia Brock, Laura Palanker Musselman

**Affiliations:** Department of Biological Sciences, Binghamton University, Binghamton, New York 13902

**Keywords:** Drosophila, Insulin, Obesity, Diabetes, High-sugar Diet, Metabolism

## Abstract

Insulin signaling is critical for developmental growth and adult homeostasis, yet the downstream regulators of this signaling pathway are not completely understood. Using the model organism *Drosophila melanogaster*, we took a genomic approach to identify novel mediators of insulin signaling. These studies led to the identification of Meep, encoded by the gene *CG32335*. Expression of this gene is both insulin receptor- and diet-dependent. We found that Meep was specifically required in the developing fat body to tolerate a high-sugar diet (HSD). Meep is not essential on a control diet, but when reared on an HSD, knockdown of *meep* causes hyperglycemia, reduced growth, developmental delay, pupal lethality, and reduced longevity. These phenotypes stem in part from Meep’s role in promoting insulin sensitivity and protein stability. This work suggests a critical role for protein homeostasis in development during overnutrition. Because Meep is conserved and obesity-associated in mammals, future studies on Meep may help to understand the role of proteostasis in insulin-resistant type 2 diabetes.

Despite the prevalence and increasing rate in incidence of T2D (Ogurtsova *et al.* 2017; Bullard *et al.* 2018), there are still holes in our understanding of the insulin signaling pathway. In both Drosophila and mammals, insulin signaling promotes glucose catabolism and lipogenesis via a highly conserved insulin receptor (InR) (Warr *et al.* 2018). As in humans, the InR is a receptor with tyrosine kinase activity that is expressed in most tissues (Wei *et al.* 2016) and signals through Akt (-Ak strain transforming, also called protein kinase B (PKB)) and the TOR (Target of rapamycin) complex (Yu *et al.* 2015). Reducing insulin-like peptide secretion or InR activity leads to hyperglycemic, insulin-resistant larvae and flies (Rulifson *et al.* 2002; Song *et al.* 2010; Zhang *et al.* 2011; Das *et al.* 2014; Musselman *et al.* 2017). Drosophila with reduced insulin-like peptide or InR activity also experience developmental delay, decreased overall growth, and often, increases in lifespan (Chen *et al.* 1996; Böhni *et al.* 1999; Tatar *et al.* 2001; Rulifson *et al.* 2002; Broughton *et al.* 2005; Shingleton *et al.* 2005; Grönke *et al.* 2010; Das *et al.* 2014; Wakabayashi *et al.* 2016; Musselman *et al.* 2017; Henstridge *et al.* 2018). Disruption of the insulin signaling pathway can therefore cause widespread physiological dysfunction in flies as in humans.

Using the genetically tractable model organism, *Drosophila melanogaster*, we previously analyzed the differentially expressed genes downstream of the InR. We focused on the fat body, which is a nutrient sensor and energy reservoir that also performs endocrine and immune functions (Na *et al.* 2013; Yongmei Xi and Xi 2015). The fat body is an organ of special interest because it develops both obesity and insulin resistance when Drosophila are reared on a high-sugar diet (HSD) (Musselman *et al.* 2011, 2013; Pasco and Léopold 2012). Previous studies in our lab have shown that fat body-specific knockdown of the InR can regulate whole-animal physiology during overnutrition. The mechanisms underlying the role of fat body InR in systemic fitness remain poorly understood. We hypothesized that the gene *CG32335*, whose expression was tightly correlated to fat body insulin signaling (Musselman *et al.* 2017), might play a role. This gene, that we have named *meep*, is intriguing but poorly studied with highly conserved orthologs in humans, rodents, frogs, fish, and nematodes, but not yeast or *Arabidopsis*. Prior to this study, the only published studies on any ortholog of Meep, showed that the human ortholog PTD012 exhibits ester hydrolase activity *in vitro* (Manjasetty *et al.* 2006). In addition, we noticed that expression of the mouse ortholog *4931406C07Rik* was positively correlated with obesity in insulin-resistant mice (Flowers *et al.* 2007). In the current study, we found that *meep* was specifically required in the HS-fed fat body to tolerate overnutrition. Meep seems to regulate insulin signaling via control of protein homeostasis, or proteostasis. This study takes the first steps in the characterization of the protein encoded by the gene *meep*.

## Materials and Methods

### Fly stocks

The control strain *w^1118^* (stock line number 60000) and *UAS-CG32335*^*RNAi*^ (*meep*^i^) (number 44172) were purchased from the Vienna *Drosophila* Resource Center. The *r4-GAL4* line was from the lab of Jae Park (Lee and Park 2004), and the *UAS-Dcr2* line was used to amplify RNAi (Dietzl *et al.* 2007). *UAS-CG32335*^*RNAi*^ (*meep*^i^) was crossed with *UAS-Dcr2*; *r4-GAL4* to produce the r4>*meep*^i^ RNAi flies, and *w^1118^* was crossed with *UAS-Dcr2*; *r4-GAL4* to produce controls. Larvae were reared from egg lay until wandering third instar and harvested from the vial wall for all experiments except for pupariation, eclosion, and longevity studies.

### Fly diet preparation

Fly stocks were maintained on standard lab food containing yeast, 5% dextrose, cornmeal, and agar. For all experiments the 0.15 M sucrose control diet was a modified Bloomington semi-defined medium containing 5% sucrose, 8% yeast, 2% yeast extract, 2% peptone and 1% agar. To produce a 0.7 M sucrose high-sugar diet (HSD) the sucrose concentration was increased to 24%. A 1.0 M sucrose diet was made by increasing the sucrose concentration to 34% (Musselman *et al.* 2011). Bortezomib and TUDCA diets contained 250 nM bortezomib (Adipogen; AG-CR1-3602-M005) or 15 mM TUDCA (VWR; 102636-650), using DMSO as a vehicle in control 0.7 M sucrose HSDs for these experiments. Cycloheximide diet contained 15 mM cycloheximide (Sigma-Aldrich; 01810-1G). Concentrations for bortezomib (Velentzas *et al.* 2013; Tsakiri *et al.* 2017), TUDCA (Debattisti *et al.* 2014), and cycloheximide (Marcos *et al.* 1982) were adapted from previous work with the added consideration of the increased stress of an HSD.

### Larval fat body collection

Wandering third instar larvae were collected from the vial wall and rinsed. The back ends of the larvae were cut off with a razor blade to gain access to the fat body. The mouth of the larvae was pinched with one hand as the cuticle was pulled toward the mouth with the other, inverting the cuticle and turning the larvae inside-out. Ten inverted larvae were then placed in a microcentrifuge tube containing 1 mL of PBS. The organs were sheared off by pipetting up and down with a P1000 pipette. Larvae were centrifuged for 1 min and then the top white layer of the pellet was suctioned off, along with all the remaining liquid and floating materials, and put on a slide. All non-fat body organs were removed from the slide leaving just the fat body to be collected into another microcentrifuge tube. Fat bodies were then centrifuged for another minute, had the supernatant removed to leave 100-200 µL of liquid, and frozen until used for experimentation.

### RNA isolation and quantitation

Fat bodies for RT and qPCR were harvested as described above with the exception that they were collected in groups of 20 rather than 10. 100 µL of fat body in PBS were mixed with 800 µL Ribozol (VWR; N580-100ML) and RNA was prepared according to the vendor’s instructions. RNA was treated with DNase (VWR; PIER89836) before reverse transcription (Bio-Rad; 1708890) and quantitative PCR (Bio-Rad; 1725270) using either *meep*-specific primers (*meep*-F: CTCTCGGAACTGAAAAGAG; *meep*-R: AACTGGGAGTCCCTTAAATC) or *Akt*-specific primers (*Akt*-F: GAGAGAGTGTGGAGTTGACG; *Akt*-R: CCATGTCTCCTTGGTAGCTG) and control primers (*rp49*-F: GCACTCTGTTGTCGATACCC; *rp49*-R: CAGCATACAGGCCCAAGAT) which recognize *rp49*, a ribosomal protein-coding mRNA that we used as a control. Each primer set extended over an intron and melting curve analyses showed a single product in each sample.

### Hemolymph glucose

Hemolymph was collected and assayed as described (Musselman *et al.* 2011). Hemolymph was pooled from 5-8 larvae to obtain 1 µL for assay. Glucose was measured by adding to 99 μl of Thermo Infinity Glucose Reagent (Fisher Scientific; TR15321) in a 96-well plate using a spectrophotometer at 340 nm (VERSA max microplate reader).

### Larval weights

Larvae were collected, rinsed with PBS, and dried on a Kim wipe prior to being collected into groups of 6 in microcentrifuge tubes and weighed on a scale.

### Triacylglycerol (TAG) assay

Total triglycerides were assayed as described previously (Musselman *et al.* 2011). Six larvae per replicate were frozen at -80 °C, then homogenized in PBS + 0.1% Tween. 2 μl of this homogenate was mixed with 198 μl of Thermo Infinity Triglyceride Reagent (Fisher Scientific; TR22421) in a 96-well plate using a spectrophotometer at 540 nm (VERSAmax microplate reader).

### Pupariation and eclosion

Adults were set on either a control or experimental diet and allowed 3 days for egg laying before the vials were cleared. To accommodate the developmental delay observed in some genotypes and diets, vials were checked at the same time daily for up to 17 days after egg lay to quantify how many larvae had reached pupariation and how many pupae eclosed into adults. Adults were removed daily to ensure they were not counted more than once.

### Longevity and survival

After eclosion, flies were given 3 days to mate before dividing males and females. Vials were checked daily to assure food was not too old or dry and were replaced accordingly. Flies were quantified every 5 days for 100 days. The same was done for the cycloheximide survival assay with the only difference being that flies were checked daily for 7 days.

### Insulin stimulation and Western blotting

Insulin stimulation experiments were performed as previously described (Musselman *et al.* 2011). Fat bodies for insulin stimulation were harvested as described above with the exception that prior to their shearing and removal, they were incubated in 1.0 μM recombinant human insulin (Sigma-Aldrich; I0259) or dilution buffer (10 mM HEPES) in oxygenated Schneider’s medium for fifteen minutes at room temperature. Fat bodies used for basal protein levels did not undergo any incubation period. Harvested fat bodies were frozen in sample buffer at -20 °C prior to immunoblotting. All samples were run on Stain-Free gels (Bio-Rad; 456-8126) allowing for total protein imaging prior to transfer. Western blotting was performed comparing both stimulated and non-stimulated samples of both control and r4>*meep*^i^ larval fat bodies. Akt band intensities were normalized to syntaxin as a loading control. Cell Signaling antibodies against *Drosophila* PO_4_-Akt (#4054) or pan-Akt (#4691) were used to detect Akt, and a U. of Iowa Developmental Studies Hybridoma Bank antibody was used against syntaxin (8C-3). Secondary antibodies were from Santa Cruz. Imaging was done using a Bio-Rad Chemidoc imager, and analyses of those images were done using the accompanying Bio-Rad Image Lab software.

### Protein quantification

Fat bodies were collected as described above with the exception that each sample was only reduced to 200-250 µL. 180 µL of sample was used for DNA isolation and quantification and the remainder was used to assay for protein concentration using Bradford’s reagent (VWR; M172). Protein concentration was measured using a spectrophotometer at 595 nm (VERSA max microplate reader). DNA was isolated using the DNeasy Blood and Tissue Kit (Qiagen; 69504). Isolation was performed according to the vendor’s instructions. Quantification of DNA was performed using the Qubit dsDNA BR Assay kit (Fisher Scientific; Q32850) with a Qubit 2.0 Fluorometer (Fisher Scientific; Q32866). Quantification was performed according to the vendor’s instructions. Protein concentrations were normalized to DNA concentrations.

### LysoTracker assay

Wandering third-instar larvae were dissected in PBS. The fat body was removed and stained with LysoTracker Red DND-99 (Thermo Fisher Scientific; L7528) diluted 1:200 to produce a final concentration of 5 μM in PBS for 2 min. Samples were immediately mounted in PBS and imaged by confocal microscopy at 543 nm. Size and number of puncta were quantified using ImageJ software, averaging the analyses of 6 representative images per larva. Each larva was one biological replicate.

### Esterase activity

Esterase activity assay was adapted from the work of Długołecka *et al.* (Długołecka *et al.* 2009). Fat bodies for esterase experiments were collected as described above, then stored in PBS buffer at -80 °C in groups of 10 fat bodies per biological replicate. Activity was determined by measuring the hydrolysis of *p*-nitrophenyl acetate (*p*NPAc) into *p*-nitrophenol (*p*NP) using a spectrophotometer at 405 nm (VERSA max microplate reader). Ten mg/mL stocks of *p*NPAc (TCI; A0040-25G) and *p*NP (Sigma-Aldrich; 1048-5G) were prepared in DMF. 500 μl of the *p*NPAc solution was combined with 9.5 mL of 50 mM Tris HCL buffer (pH = 7.4) to make a working substrate solution. Five μl of the *p*NP solution was added to 495 μl of the substrate solution to make the standard curve for quantification. Fat bodies were assayed in a 96-well plate by adding 175 μl of substrate solution to sample wells containing 25 μl of homogenized fat body in unison with a multi-channel pipette. A reading was immediately taken upon the addition of the substrate solution and again every minute for 5 min. The time zero measurement was subtracted from all other measurements to account for the absorbance of the sample itself. Corrected absorbances were then used to calculate the amount of product produced per minute, giving five rate measurements per sample. These values were averaged to give one final data point for each sample. Esterase activity was normalized to total protein using Bradford’s reagent (VWR; M172).

### Statistical analysis

GraphPad PRISM 8 software was used to plot data and perform statistical analysis. Pairwise comparison *P*-values involving only 2 groups were calculated using a Student’s two-tailed *t*-test. Pairwise comparison *P*-values involving more than 2 groups were calculated using a one-way ANOVA with multiple comparison tests for post-hoc analysis. Survival-style curve comparison *P* and χ^2^-values were calculated using the Mantel-Cox log-rank test. Error bars represent SEM.

### Data availability

All strains used are available through the Vienna *Drosophila* Resource Center. A supplementary material file in the online of this article contains Figures S1 and S2 and Table S1. Supplemental material available at figshare: https://doi.org/10.25387/g3.13023215.

## Results

### Expression of meep is insulin receptor- and sugar-dependent

The gene *meep* (*CG32335*) was chosen from a list of 220 genes predicted to be downstream of insulin signaling in a previous study (Musselman *et al.* 2017). *Meep* was differentially expressed depending on both InR expression and dietary sugar content in larval fat bodies ((Musselman *et al.* 2017) see supplementary dataset). InR activation, via expressing a constitutively active transgenic A1325D InR, increased expression of *meep* 16.7-fold in fat bodies, compared to controls (Musselman *et al.* 2017). InR knockdown using fat body-specific transgenic RNA interference (RNAi) decreased *meep* expression 26.9-fold compared to controls (Musselman *et al.* 2017). Using RT and qPCR, we found that increasing dietary sugar content, which reduces insulin signaling (Musselman *et al.* 2011; Pasco and Léopold 2012), also decreased fat body *meep* expression (Figure S1), confirming previous RNA-seq studies (Musselman *et al.* 2017). Therefore, we hypothesized that Meep might function in HSD-induced insulin resistance. Because *meep* was differentially expressed in insulin resistant fat bodies, we used an RNAi strategy to knock it down in the larval fat body.

### Meep is required in order to tolerate an HSD

Fat body expression of a *UAS-CG32335*^*RNAi*^ transgene was achieved using the *r4-GAL4* driver. The *UAS-**CG32335*^*RNAi*^ construct used was the only line available for this target gene and had no predicted off-targets. qPCR results quantifying *meep* mRNA from larval fat bodies showed 75.0% knockdown when reared on a control diet and 45.9% knockdown when reared on an HSD, when compared to genotypically-matched controls made from the GAL4 drivers crossed to the *w^1118^* genetic background into which the RNAi transgene was introduced, hereafter called the “control” genotype (Figure S1). We also saw a reduction in fat body *meep* mRNA after HS feeding, confirming what was seen in previous RNA-seq studies (Figure S1) (Musselman *et al.* 2017). The r4>*meep*^i^ larvae were tested for insulin-like phenotypes due to the differential expression of *meep* in InR transgenic fat bodies. When *meep* was knocked down in the larval fat body, larvae were able to survive in large numbers when reared on our control diet, which has 0.15 M sucrose. This was not true when the sugar concentration was increased to 1.0 M sucrose. Knocking down *meep* in the larval fat body led to few larvae reaching the third instar, with none surviving to pupariation (data not shown). For this reason, we lowered the concentration of sucrose to 0.7 M, which allowed the survival of some r4>*meep*^i^ larvae. We will be referring to this concentration as an HSD.

**Figure 1:**
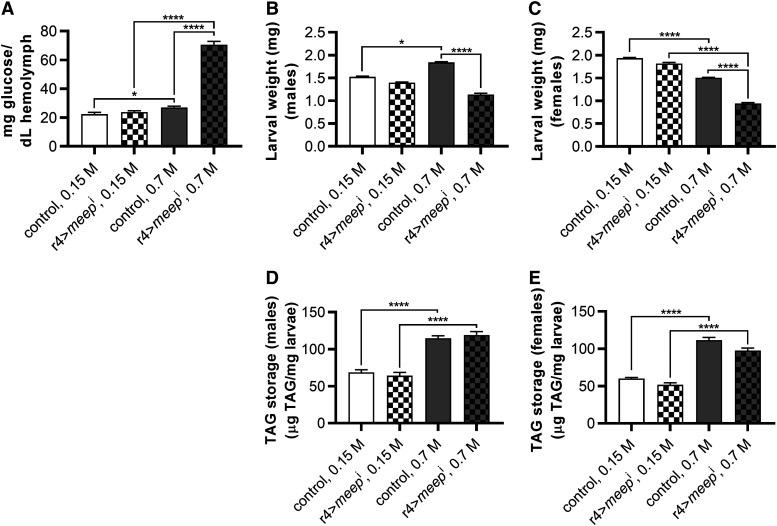
Meep is protective against HSD-induced elevated hemolymph glucose and reduction in growth. Larvae were reared on either a control diet or an HSD until the post-feeding wandering third instar stage where they were assayed for their hemolymph-glucose concentrations (A; n ≥ 30 per experimental group) after being weighed in male (B) and female (C) groups of 6 (n ≥ 16 per experimental group). Larvae were assayed for TAG content, normalized to larval mass, in male (D) and female (E) groups (n ≥ 16 per experimental group). Error bars represent SEM, *P < 0.05; ****P < 0.0001.

When *meep* was knocked down in larvae fed a control diet, there was no difference in hemolymph glucose levels when compared to controls (Figure 1A). When reared on an HSD, r4>*meep*^i^ larvae experienced a 2.6-fold increase in hemolymph glucose concentrations compared to controls (Figure 1A). Overall animal weights in r4>*meep*^i^ larvae also displayed severe phenotypes on an HSD compared to control diets. There was no difference when reared on a control diet, and a decrease of 38.4% and 37.5% when reared on an HSD in male and female r4>*meep*^i^ larvae respectively when compared to controls (Figure 1B,C). Thus, *meep* is required to maintain hemolymph glucose levels as well as normal weight.

High-sugar feeding has been associated with obesity, which was quantified as total triacylglycerol (TAG) content per unit mass. As in previous studies, an HSD increased the relative fat mass in stage-matched larvae (Figure 1D,E). This was true in the control genotype (67.3% increase in males, 86.3% in females) and in r4>*meep*^i^ larvae (85.5% in males, 88.2% in females). Despite the large effects of *meep* knockdown on size, there was no change in relative TAG concentration.

### Meep is required for development and survival in larvae reared on an HSD

**Figure 2:**
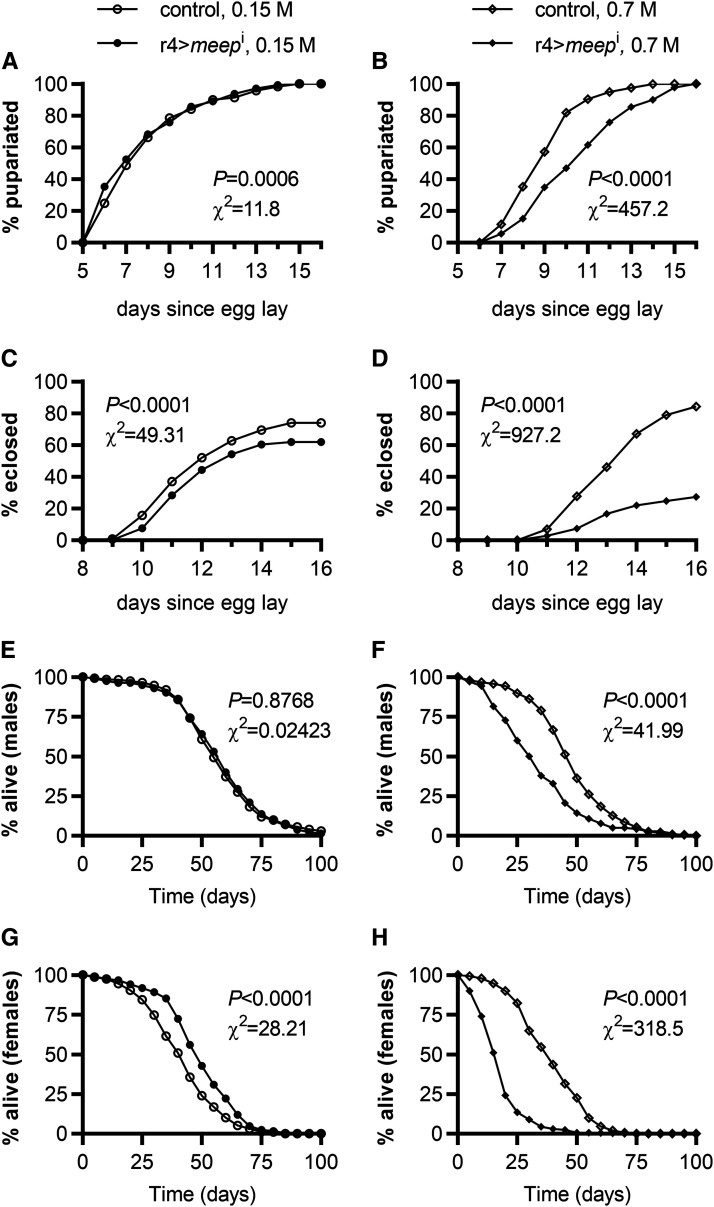
Meep is required for development and survival on an HSD. Reared on either a control or an HSD, larvae were observed from egg lay up to pupariation (A-B; n > 1000 per experimental group), and observed from pupariation to eclosion into adults (C-D; n > 300 per experimental group). Adults were then separated between males and females and observed for 100 days (E-H; n ≥ 140 per experimental group).

Because both an increase in hemolymph glucose and a decrease in weight are associated with reduced insulin signaling, we looked at additional insulin-dependent phenotypes, developmental rate and longevity (Chen *et al.* 1996; Tatar *et al.* 2001; Shingleton *et al.* 2005; Bai *et al.* 2013; Musselman *et al.* 2017). We observed the rate at which larvae reached pupariation and the rate at which those pupae eclosed into adults. Due to the high n in this line of experimentation even the smallest of differences were recognized as statistically significant, and for this reason we not only report the *P* value but also the χ^2^ value in order to better describe the degree of difference between r4>*meep*^i^ and controls. There was a slight difference between the rate of pupariation of r4>*meep*^i^ and controls when reared on a control diet (*P* = 0.0006; χ^2^ = 11.8) (Figure 2A). However, similar to the aforementioned experiments, r4>*meep*^i^ flies produced a more severe phenotype when reared on an HSD. Controls reached 50% pupariation by 9 days after egg laying, while it took r4>*meep*^i^ larvae 11 days, showing a dramatic 22.5% and 28.9% delay in development time 9 and 11 days after egg laying respectively (*P* < 0.0001; χ^2^ = 457.2) (Figure 2B). Knocking down *meep* not only delayed the rate of pupariation, but also had a negative effect on pupariation success. When reared on a control diet, 73.9% of control pupae eclose into adults while 61.9% of r4>*meep*^i^ pupae eclose into adults, showing a significant difference even in the absence of an HSD (*P* < 0.0001; χ^2^ = 49.31) (Figure 2C). When reared on an HSD, the difference in eclosion rates are more severely affected, with 84.3% of control pupae reaching adulthood and only 27.4% of r4>*meep*^i^ pupae reaching adulthood (*P* < 0.0001; χ^2^ = 927.2) (Figure 2D).

Interested to find out if this reduction in survival persists through adulthood, we conducted a survival assay that lasted 100 days. When reared on a control diet there was no noticeable difference between male r4>*meep*^i^ and control flies, however, female r4>*meep*^i^ flies were surviving longer than controls by a week (Figure 2E,G). The median survival time for female r4>*meep*^i^ flies was 47 days, and for controls was 40 days when reared on a control diet (*P* < 0.0001; χ^2^ = 28.21) (Figure 2G). By contrast, the median survival time for male control flies reared on an HSD was 45 days, while for r4>*meep*^i^ flies it was only 30 days (*P* < 0.0001; χ^2^ = 41.99) (Figure 2F). Similar, but far more severe, female control flies reared on an HSD had a median survival time of 37 days while r4>*meep*^i^ females only had 15 days, the shortest of the four experimental groups (*P* < 0.0001; χ^2^ = 318.5) (Figure 2H). Increased longevity, and developmental delay are reminiscent of phenotypes in insulin-resistant Drosophila (Tatar *et al.* 2001; Shingleton *et al.* 2005; Musselman *et al.* 2017), leading us to test Meep’s effect on insulin sensitivity.

### Meep maintains proper insulin signaling

In both mammalian systems (Guerra *et al.* 2001), and in Drosophila (Haselton *et al.* 2010), insulin resistance can be determined by the phosphorylation state of downstream components of the insulin signaling pathway. We measured insulin sensitivity in fat body collected from larvae reared on an HSD by stimulating this tissue by incubating with recombinant human insulin and quantifying phosphorylated Akt (P-Akt) at serine 505 via Western blot. Akt is an integral protein downstream of the InR that is phosphorylated at this residue downstream of ligand binding and insulin receptor activation (Verdu *et al.* 1999). This phosphorylation event is catalyzed by the TORC2 complex and is required for full activation of Akt, including insulin-dependent glucose transporter trafficking and glucose uptake (Hresko and Mueckler 2005; Breuleux *et al.* 2009; Yang *et al.* 2015). Therefore, P-Akt is a marker for insulin signaling.

**Figure 3:**
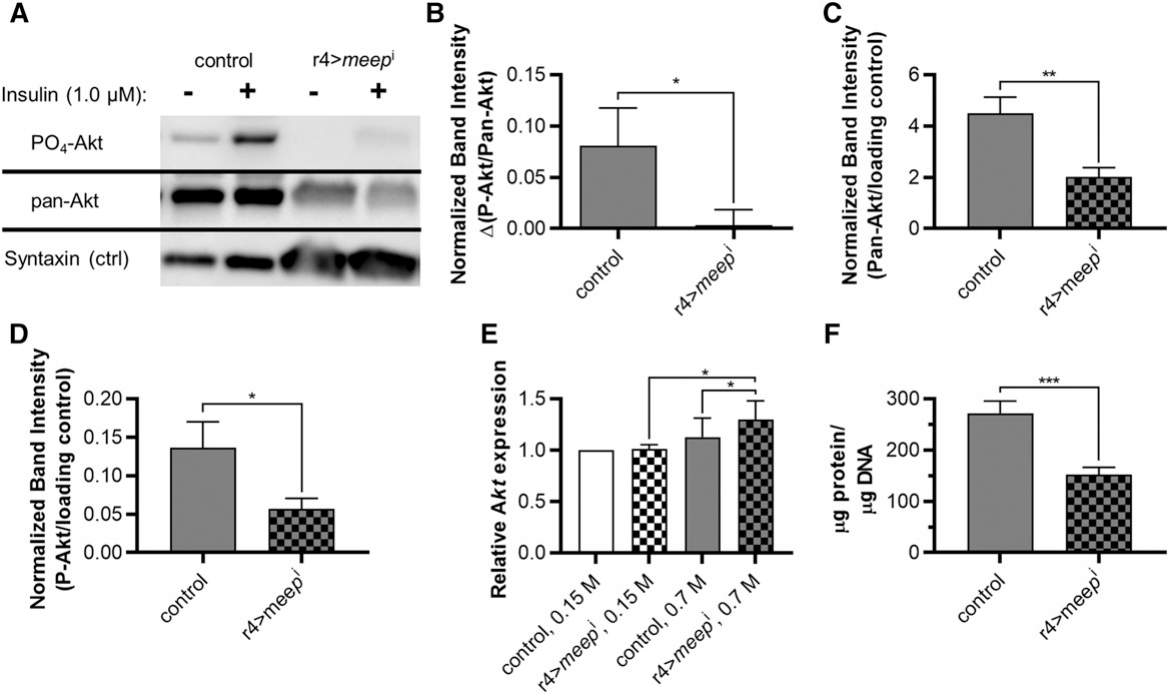
Meep may support proper insulin signaling by promoting insulin sensitivity and protecting against Akt and total protein loss. Fat bodies harvested from wandering third instar larvae either stimulated (+) or not stimulated (-) with insulin were prepared for Western blotting probing for PO_4_-Akt, and then again separately for pan-Akt, using syntaxin as a loading control (A). Data were graphed as the change in proportion of Akt phosphorylated in response to insulin stimulation (B; n = 8 per experimental group). Quantitation of pan-Akt Western blots (C; n ≥ 17 per experimental group). PO_4_-Akt Westerns were repeated on fat bodies that did not undergo any stimulation or incubation period to measure basal PO_4_-Akt (D; n = 11 per experimental group). *Akt* mRNA is increased on an HSD when *meep* is knocked down (E; n = 5 per experimental group). RT and qPCR data are graphed using the 2^-∆∆Ct^ method to show fold change (Livak and Schmittgen 2001). All statistical tests are performed on log10 transformed ∆Ct values. Total concentration of protein in fat bodies was reduced when *meep* was knocked down (F; n = 10). Error bars represent SEM, *P < 0.05; **P < 0.01; ***P < 0.001.

We performed Westerns separately probing with a P-Akt antibody and a Pan-Akt antibody that binds both phosphorylated and non-phosphorylated Akt (Figure 3A). Insulin sensitivity was expressed as the change in ratio of phosphorylated Akt in response to insulin stimulation. Control fat bodies were significantly more sensitive to insulin increasing the relative Akt phosphorylation ratio by 0.0808, whereas it only increased 0.00329 in r4>*meep*^i^ fat bodies (Figure 3B). Pan-Akt Westerns also revealed that r4>*meep*^i^ fat bodies suffered from a 52.1% decrease in total Akt (Figure 3C). We also repeated the Westerns in an insulin-independent experiment without an incubation period to quantify the effect on basal levels of P-Akt in the fat body. We found basal P-Akt was 58.3% lower in r4>*meep*^i^ than control fat bodies (Figure 3D), which was very similar to the decrease in total Akt. These Westerns reveal that not only are r4>*meep*^i^ fat bodies suffering from a decrease in Akt but that Akt is not phosphorylated sufficiently in response to insulin. This would significantly reduce the fat body’s ability to perform insulin signaling and suggests that Meep either reduces Akt degradation or promotes *Akt* expression. To address this, we measured *Akt* expression by isolating RNA and performing RT-qPCR. There was no difference in *Akt* expression on a control diet, but there was a 15.1% increase in r4>*meep*^i^ fat bodies compared to controls fed an HSD (Figure 3E). This suggests Meep may be required for protein stability. Therefore, we tested relative protein content and found a 44.1% decrease in r4>*meep*^i^ fat bodies (Figure 3F). These data suggest a role for Meep in proteostasis and suggest that the phenotypes observed in HS-fed r4>*meep*^i^ could in part result from an increase in protein turnover. Misfolded and unfolded proteins that are not brought to their correctly folded state do not pass endoplasmic reticulum (ER) quality control and are broken down via protein turnover, which occurs in the proteasome or lysosome (Arsham and Neufeld 2009; Kang and Ryoo 2009). Therefore, to further explore proteostasis in r4>*meep*^i^ fat bodies, we first investigated Meep’s effect on autophagy.

### Reducing Meep leads to a reduction in organelle acidification

**Figure 4:**
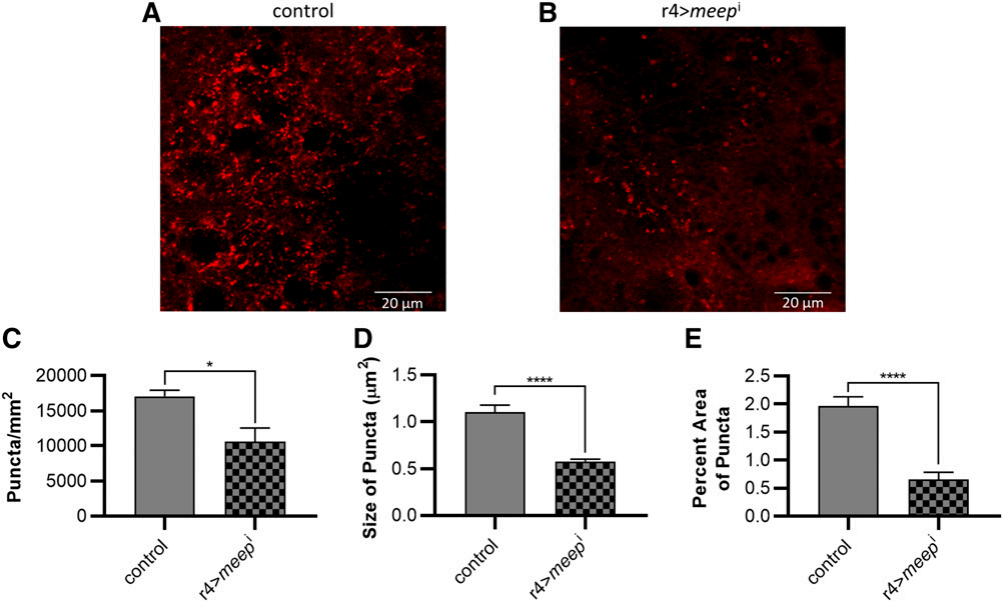
Meep is required for proper organelle acidification on an HSD. Fat bodies harvested from wandering third instar larvae were stained using the acidotropic dye LysoTracker Red and imaged via confocal microscopy (A-B). Images were analyzed for the number of puncta (C), size of puncta (D), and percent area of tissue covered by puncta (E). Error bars represent SEM (n ≥ 10 per experimental group), *P < 0.05; ****P < 0.0001.

The lysosomal degradation pathway enables the digestion of proteins via a low-pH process called autophagy (Xu and Ren 2015). To measure acidic organelles in larval fat bodies, we stained them with the acidotropic dye LysoTracker Red, a marker for lysosome/autophagy activity. This dye stains acidic organelles such as lysosomes, late endosomes, or autophagosomes, all of which function in autophagic protein turnover (Luzio *et al.* 2007). Imaging revealed an overall reduction in acidic organelles in r4>*meep*^i^ fat bodies (Figure 4A,B). r4>*meep*^i^ fat bodies contained 37.8% less puncta than controls (Figure 4C). Puncta found in r4>*meep*^i^ fat bodies were also 47.7% smaller than those found in controls. This reduction in puncta number and size resulted in a 66.7% reduction in area covered by puncta in r4>*meep*^i^ fat bodies when compared to controls. These data suggest that r4>*meep*^i^ fat bodies may have reduced autophagy and/or lysosomal activity. If r4>*meep*^i^ fat bodies have a defect in proteostasis, then they may also be experiencing elevated ER stress (Yang *et al.* 2010). The ER plays a key role in maintaining protein stability, participating in protein synthesis, folding, and transport. The accumulation of unfolded or misfolded proteins leads to ER stress (Ryoo *et al.* 2007; Zhang *et al.* 2013).

### Meep is hypersensitive to proteasomal inhibition

**Figure 5:**
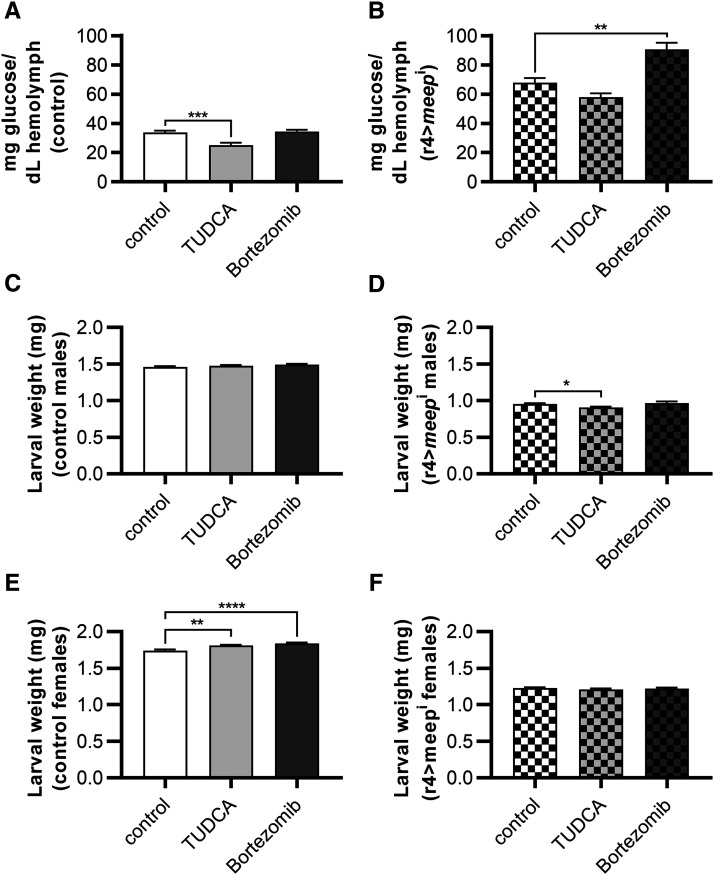
*Meep* knockdown-associated hyperglycemia is exacerbated by inhibiting the proteasome. Larvae were reared on either a control HSD with vehicle or one supplemented with TUDCA or bortezomib until the post-feeding wandering third instar stage where they were assayed for their hemolymph-glucose concentrations (A, B; n ≥ 30 per experimental group) after being weighed in male (C, D) and female (E, F) groups of 6 (n ≥ 20 per experimental group). Error bars represent SEM, *P < 0.05; **P < 0.01; ***P < 0.001; ****P < 0.0001.

Because autophagy did not appear to be increased, we attempted to stabilize the proteome by supplementing HSDs with the ER stress-reducing protein chaperone Tauroursodeoxycholic acid (TUDCA). TUDCA improved HSD-induced hyperglycemia for controls, lowering hemolymph glucose concentrations by 25.8%, but not for 4>*meep*^i^ larvae (Figure 5A,B). Adding TUDCA had a minor effect increasing control female weights by 4.02% and reducing r4>*meep*^i^ male weights by 4.81%, but had no effect on control males or r4>*meep*^i^ females (Figure 5C-F). The HS-induced larval developmental delay went unchanged for controls but was improved for r4>*meep*^i^ larvae by almost a full day (*P* > 0.0001) (Figure S2A; Table S1). To our surprise, the addition of TUDCA considerably reduced the final pupal counts for both control and r4>*meep*^i^ larvae but had no effect on eclosion (Figure S2; Table S1). Despite its effects on reducing hemolymph-glucose in controls, attempting to stabilize proteins by reducing ER stress via TUDCA did not rescue r4>*meep*^i^ larvae.

Another mechanism that could explain reduced protein content could be increased proteasomal activity (Qiu *et al.* 2019). The proteasomal pathway involves targeted degradation of intracellular proteins and plays a regulatory role in almost all basic cellular processes (Varshavsky 2005; Tanaka 2009). Therefore, we attempted to stabilize the proteome using the proteasomal inhibitor bortezomib (Chen *et al.* 2011; Tsakiri *et al.* 2017). In contrast to TUDCA, bortezomib affected hemolymph glucose concentrations in r4>*meep*^i^ larvae and not controls, increasing them 33.7% (Figure 5B). Bortezomib had a modest effect on control female weights increasing them 5.65% as well as increasing developmental timing of controls by 0.6 days (Figures 5E and S2A; Table S1). Pupal lethality was exacerbated in r4>*meep*^i^ larvae fed bortezomib, resulting in almost no larvae eclosing as well as a delay for the few that did (*P* > 0.0001) (Figure S2B; Table S1). In contrast to the increase in growth of female controls, bortezomib only produced negative effects in the r4>*meep*^i^ genotypes, revealing a sensitivity to proteasomal inhibition in r4>*meep*^i^ animals in the form of further increased hemolymph glucose concentrations (Figure 5B) and pupal lethality (Figure S2B, Table S1).

**Figure 6:**
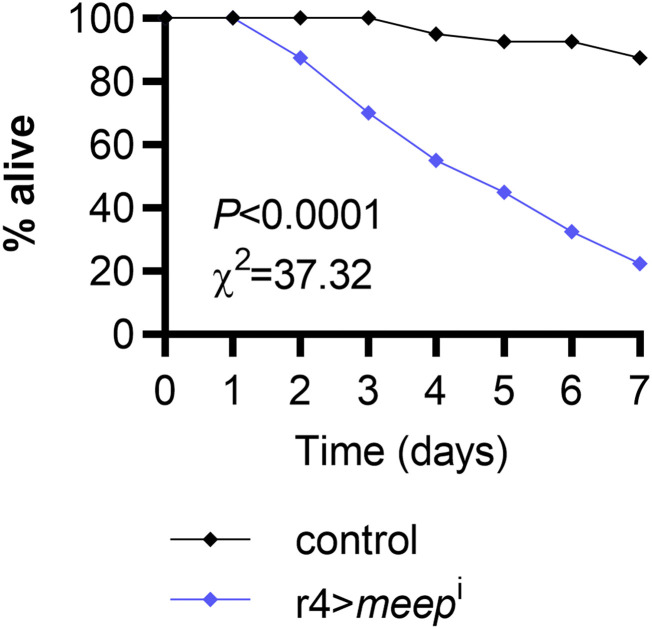
*Meep* knockdown animals are sensitive to translation inhibition via cycloheximide. Adults reared on an HSD were fed a diet supplemented with cycloheximide to assess survival (F; n = 60 per experimental group).

Another potential contributor to Meep loss-of-function proteostasis defects may be insufficient translation. To test this, we reared larvae on an HSD until eclosion, when adults were transferred to an HSD supplemented with the translation inhibitor cycloheximide. After seven days, 12.5% of controls died, whereas 77.5% of r4>*meep*^i^ flies died (Figure 6; *P* > 0.0001). These data demonstrate that HS-fed r4>*meep*^i^ animals exhibit hypersensitivity to translation inhibition as well as proteasomal inhibition, compared with the control genotype.

### Meep supports esterase activity against a synthetic ester substrate

**Figure 7:**
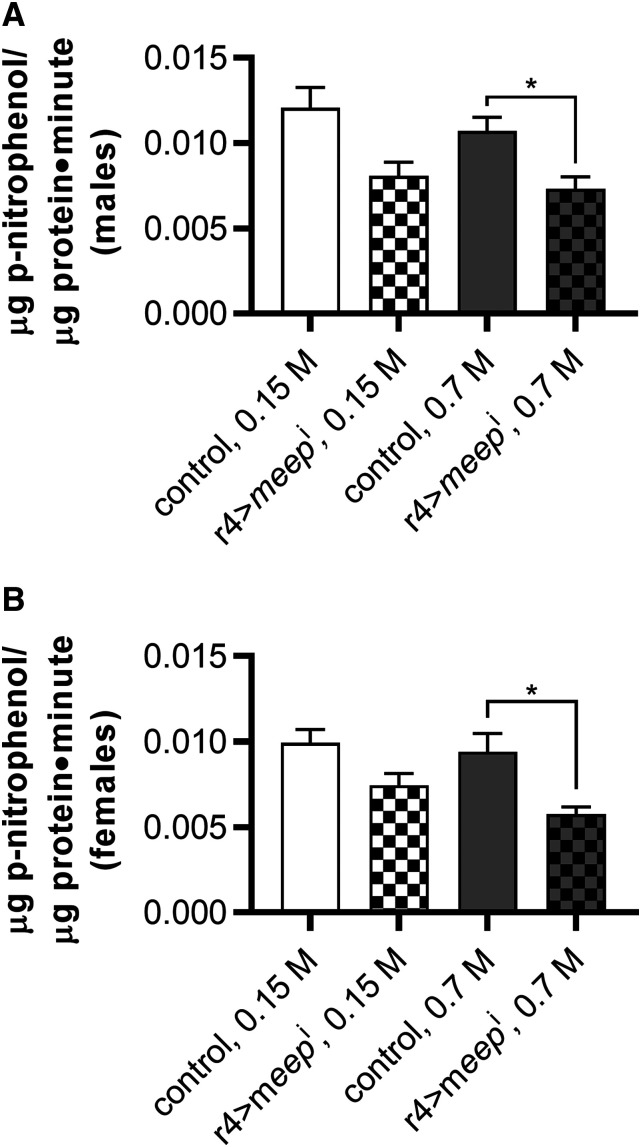
Meep supports esterase activity against a synthetic ester substrate on an HSD. Homogenized fat bodies were incubated with p-nitrophenyl acetate for five minutes while the production of p-nitrophenol was measured colorimetrically every minute in both (A) males, and (B) females. Error bars represent SEM (n = 14 per experimental group), *P < 0.05.

Meep’s human ortholog, PTD012, has been shown to exhibit ester hydrolase activity against a synthetic ester substrate (Manjasetty *et al.* 2006). Therefore, we performed an esterase activity assay by colorimetrically quantifying the rate at which fat bodies were able to convert the synthetic ester *p*-nitrophenyl acetate into *p*-nitrophenol. There was no difference between the rates of ester hydrolysis of controls and r4>*meep*^i^ fat bodies when reared on a control diet. When reared on an HSD, r4>*meep*^i^ fat bodies experienced a decrease in ester hydrolysis rate of 31.7% and 38.7% among males and females respectively, when compared to controls (Figure 7). These results suggest that *meep* may support ester hydrolase activity. This effect was small considering the dramatic reduction in fat body *meep* mRNA level after HS rearing (Figure S1), and the severe phenotypes observed in r4>*meep*^i^ animals.

## Discussion

In this study, we aimed to characterize the function of the novel gene *meep*. Knocking down *meep* in larval fat bodies resulted in phenotypes similar to that of fat body InR knockdown (Musselman *et al.* 2017), consistent with a proposed role in insulin signaling. All r4>*meep*^i^ phenotypes tested were stronger when larvae were reared on an HSD, which is when *meep* expression is at its lowest. This leads us to hypothesize that a minimum threshold of required Meep function isn’t being met, resulting in negative physiological consequences. Meep seems to support the catalysis of ester hydrolysis and has a surprising role in maintaining protein stability, including that of the integral insulin signaling pathway kinase Akt.

The insulin signaling pathway kinase Akt plays several roles consistent with the observed r4>*meep*^i^ growth phenotypes. This cytoplasmic signaling protein, when activated by phosphorylation, binds to and phosphorylates many conserved substrates affecting an array of cellular functions (Manning and Toker 2017). Akt promotes glucose uptake through plasma membrane-targeted translocation of glucose transporters (Calera *et al.* 1998) and also promotes cell and organismal growth (Rommel *et al.* 1999; Sarbassov *et al.* 2005; Wullschleger *et al.* 2006) and lifespan (Brunet *et al.* 1999; Datta *et al.* 1999). The reduction in Akt as well as its response to insulin (Figure 3B-C) in r4>*meep*^i^ fat bodies could therefore partially explain the increase in hemolymph glucose (Figure 1A), decrease in larval weight (Figure 1B,C), developmental delay (Figure 2A-D), and reduction in longevity (Figure 2E-H) when reared on an HSD. Interestingly, fat bodies from r4>*meep*^i^ larvae contained increased Akt mRNA (Figure 3E), suggesting there may be an attempt by the fat body to compensate for reduced Akt activity at the transcriptional level.

Proper cellular function is sustained by maintaining a healthy proteome; this requires a balance between protein synthesis and turnover. Protein synthesis requires amino acids, ribosomal subunits to put them together, and enough energy to do so. Insulin has been shown to increase the rate and overall amount of protein synthesis (Rulifson *et al.* 2002; Essers *et al.* 2016) and ribosomal activity (Grewal *et al.* 2007; Essers *et al.* 2016) but has a more complex relationship with proteostasis. Because our insulin-resistant r4>*meep*^i^ fat bodies had reduced protein content (Figure 3F), we hypothesized that the mechanism underlying insulin resistance could in part be a reduced amount of the proteins required for insulin signaling. Starting with lysosomal protein degradation, we probed the roles of organelles involved in protein stability and found fewer acidic organelles in r4>*meep*^i^ fat bodies (Figure 4), which could correspond to reduced lysosomal activity. Reduced lysosomal activity has been shown to reduce insulin sensitivity and increase ER stress (Yang *et al.* 2010), so we added the ER stress-reducing protein chaperone TUDCA to an HSD. TUDCA improved hemolymph glucose in controls (25.8% reduction) but not in r4>*meep*^i^ larvae (Figure 5A,B). This is consistent with reduced hemolymph glucose in PBA-supplemented, HS-fed flies (Musselman *et al.* 2017) and also with previous studies showing that reducing ER stress with chemical chaperones improves hyperglycemia in mouse T2D models (Ozcan *et al.* 2004, 2006; Guo *et al.* 2015). However, adding TUDCA to HS feeding reduced pupariation success for both genotypes (Figure S2A; Table S1). Although TUDCA has been shown to improve cellular stress associated with the unfolded protein response, it also affects digestion (Portincasa *et al.* 1993) and the microbiome (Wang *et al.* 2018). Therefore, this drug is likely to play a complex role in diet-induced insulin resistance.

A reduction in protein in r4>*meep*^i^ could be due to increased protein turnover via the proteasome, so we supplemented the HSD with the proteasomal inhibitor bortezomib. This was expected to improve physiology by increasing protein content and stability. However, the opposite result was observed: bortezomib reduced the health of HSD-fed r4>*meep*^i^ larvae even further by increasing hemolymph-glucose concentrations as well as pupal lethality (Figures 5B and S2B; Table S1). Our data suggests that reduced protein and acidic organelle content in r4>*meep*^i^ fat bodies is associated with hypersensitivity to proteasomal inhibition, consistent with a model where the proteasome is even more important when Meep is reduced. A reduction in autophagy can be compensated for by an increase in proteasomal activity and vice versa (Qiu *et al.* 2019), supporting this model. Considering that insulin signaling can increase translation (Proud and Denton 1997), we utilized the translation inhibitor cycloheximide to reduce the production of proteins. Supplementing an HSD with cycloheximide resulted in the death of 65% more r4>*meep*^i^ than it did control flies (Figure 6). This hypersensitivity to cycloheximide is consistent with a model where insulin-resistant r4>*meep*^i^ fat bodies already suffer from reduced translation to such a degree that further inhibition is deadly. Reduced translation has been linked to insulin resistance in other studies (Essers *et al.* 2016; James *et al.* 2017) and could arise due to defects in amino acid import, aminoacyl tRNA synthesis, or ribosome function. Such changes would reduce the need for autophagy and would result in an overall reduction in many fat body proteins in r4>*meep*^i^ animals, not just Akt. Future studies will probe the role of Meep in regulating other proteins in the insulin signaling pathway and other pathways associated with development and longevity.

Interestingly, r4-*meep*^i^ phenotypes were most pronounced during metamorphosis, as most of these animals complete pupariation (albeit delayed) but few eclose to reach adulthood (Figure 2B,D; Table S1). Autophagy is required for Drosophila metamorphosis (Lee and Baehrecke 2001), and reducing autophagy is detrimental to eclosion success (Mulakkal *et al.* 2014). Considering that r4>*meep*^i^ larvae appear to suffer from reduced autophagy (Figure 4), it is not surprising that we observed a lower survival of larvae and pupae in this genotype. TUDCA also reduces apoptosis (Rodrigues *et al.* 2003; Miller *et al.* 2007), which is an important function during tissue patterning (Rusconi *et al.* 2000) and metamorphosis (Jiang *et al.* 2000; Wang *et al.* 2008; Kang and Bashirullah 2014). TUDCA may have improved glucose tolerance in controls via amelioration of HS-induced ER stress in larvae, whereas TUDCA’s reduction of apoptosis impaired pupal development, leading to fewer pupae and therefore adult flies in both genotypes (Figures 5A and S2A; Table S1). Considering the strongest Meep-specific phenotype produced from TUDCA feeding was a reduction in developmental delay, these results suggest that Meep may not directly influence ER stress during overnutrition, but perhaps contributes via its role in maintaining protein stability and/or apoptosis.

Despite the negative effects on survival observed for r4>*meep*^i^ flies fed an HSD, not all changes were negative on a control diet. Female r4>*meep*^i^ flies reared on our control diet experienced a dramatically extended lifespan compared to controls (Figure 2G), similar to that of female *InR* mutant flies fed control diets (Tatar *et al.* 2001). Decreased insulin signaling also extends lifespan in worms (Kimura *et al.* 1997) and mice (Blüher *et al.* 2003). Due to the pleiotropic effects of insulin, it is unclear exactly why this lifespan extension occurs, but may be related to reduced translation, which would explain the reduced protein levels seen in r4>*meep*^i^ fat bodies. Reduced translation has been observed in long-lived, insulin-deficient flies and mice (Essers *et al.* 2016) and could slow down aging via slowing cellular and organismal growth (Piper and Partridge 2018).

This study identified and characterized a novel regulator of insulin signaling required for tolerating a high-sugar diet. *Meep* is required for maintenance of glucose homeostasis, insulin sensitivity, and protein stability. Future studies will further characterize the biochemistry that makes Meep essential specifically during HS feeding. Our data suggests that there is a complex relationship between diet and Meep-dependent esterase activity, where overall esterase activity doesn’t increase, yet the demand for Meep increases on HS, possibly due to defective proteostasis. Previous research has shown that the methyl-esterase PME-1 can play a role in protein stability, improving protein half-life and activity by protecting against proteasomal degradation (Yabe *et al.* 2015). Interestingly, although we observed no changes in TAG metabolism in r4>*meep*^i^ larvae (Figure 1D,E), previous studies have shown that Meep physically interacts with the TAG lipase Brummer (Lowe *et al.* 2014), suggesting that it could function in lipolysis under different feeding conditions, such as starvation. Another protein-protein interaction study (Guruharsha *et al.* 2011) showed that Meep physically interacted with Nurf38, another recently discovered insulin signaling pathway protein (Liu *et al.* 2020). Taken together, our studies indicate that Meep is a protein that protects against diet-induced insulin resistance via a novel, proteostasis-based mechanism.
